# Why are the batteries in the microwave?: Use of semantic information under uncertainty in a search task

**DOI:** 10.1186/s41235-021-00294-1

**Published:** 2021-04-14

**Authors:** Gwendolyn L. Rehrig, Michelle Cheng, Brian C. McMahan, Rahul Shome

**Affiliations:** 1 Department of Psychology, University of California, Davis, CA 95616 USA; 2 School of Social Sciences, Nanyang Technological University, Singapore, 639798 Singapore; 3 Department of Computer Science, Rutgers University—New Brunswick, New Brunswick, USA; 4 Department of Computer Science, Rice University, Houston, USA

**Keywords:** Scene semantics, Visual search, Decision-making, Learning, Prior knowledge, Belief updating, Bayesian decision-making, Visuomotor behavior

## Abstract

A major problem in human cognition is to understand how newly acquired information and long-standing beliefs about the environment combine to make decisions and plan behaviors. Over-dependence on long-standing beliefs may be a significant source of suboptimal decision-making in unusual circumstances. While the contribution of long-standing beliefs about the environment to search in real-world scenes is well-studied, less is known about how new evidence informs search decisions, and it is unclear whether the two sources of information are used together optimally to guide search. The present study expanded on the literature on semantic guidance in visual search by modeling a Bayesian ideal observer’s use of long-standing semantic beliefs and recent experience in an active search task. The ability to adjust expectations to the task environment was simulated using the Bayesian ideal observer, and subjects’ performance was compared to ideal observers that depended on prior knowledge and recent experience to varying degrees. Target locations were either congruent with scene semantics, incongruent with what would be expected from scene semantics, or random. Half of the subjects were able to learn to search for the target in incongruent locations over repeated experimental sessions when it was optimal to do so. These results suggest that searchers can learn to prioritize recent experience over knowledge of scenes in a near-optimal fashion when it is beneficial to do so, as long as the evidence from recent experience was learnable.

## Significance statement

This study investigated how people use semantic knowledge to search for common household objects that have been hidden in unusual places within a room. The task was constructed so that long-standing world knowledge of likely locations could be useful in navigating such a context, provided that people learn to rely on recent experience when longstanding beliefs lead to poor search outcomes. Results from the present study showed that it is possible to learn to use recent experience with objects being hidden in unusual places to search more effectively, but that the ability to do so is limited. A practical implication 1 of these findings is the possibility to differentiate between deficits in long-term memory and episodic memory in older adults who have been diagnosed with dementia, as these can often be difficult to dissociate within the same task. By differentiating these two characteristic memory deficits, clinicians may gain insight as to the severity of each deficit and provide a more targeted intervention that can help improve their patient's livelihood and quality of life.

Imagine this scenario: the income tax return deadline rapidly approaches, and you have been working night and day to meet it. You find yourself in desperate need of a cup of coffee, but your trusty coffee mug is nowhere to be seen. Where do you search for it? What if a thorough search through all of the usual places—your desk, the sink, the cabinet—produces no results? Suppose you finally discover that, in your sleep-deprived state, you mistakenly tossed the mug in the trash can. Moreover, suppose this happens *repeatedly*. Would you learn that the location you once thought was least likely to contain the coffee mug is now a more probable location that you should search in the future? When given enough evidence, can we learn new expectations about where objects are likely to be in a familiar environment, even when the evidence guides you to search in otherwise *unlikely* places?

The goal of the current study was to investigate whether prior knowledge of scene semantics and recent experience can be used optimally to guide search behavior. Search was carried out within computer-illustrated environments made to resemble a kitchen and a living room. The expected locations of targets (world knowledge) were determined empirically, and then search was tested when target location probabilities were manipulated to be either congruent or incongruent with scene semantics. A condition in which target locations were selected randomly from a uniform distribution was also included. Human performance was compared to that of simulated ideal Bayesian observers that searched scenes using world knowledge, recent experience, or a combination thereof. In the active search task, points were awarded for successfully finding target objects, and points earned by human observers were compared to the simulated performance of an ideal Bayesian observer.

## Background

Scene semantics have been shown to influence gaze position and search performance when searching real-world scenes (Castelhano and Henderson 2007, 2008; Henderson et al. 2009; Vo and Henderson 2010). Viewers tend to prioritize locations that are most likely to contain targets (Torralba et al. 2006). Search for targets in locations that are congruent with scene semantics is generally faster than search for targets in incongruent locations (Castelhano and Henderson 2007; Hillstrom et al. 2012; Hollingworth 2009; Wolfe et al. 2011, 2010; Vo and Wolfe 2013a). Visual search is slower and less accurate when targets do not belong in the scene (e.g., a toaster in a playground; Henderson et al. 1999), or do not belong in a particular location (e.g., an airplane in the lower half of an image; Malcolm and Henderson 2009; Neider and Zelinsky 2006), which further suggests that scene semantics play a role in visual search. Search is impaired when semantic cues are removed by either altering (Vo and Wolfe 2013b), or scrambling (Biederman et al. 1973; Wu et al. 2014) the scene. In these studies, scene semantics are typically manipulated through violations of scene grammar: the prior expectations about the possible relationships between objects in a scene (Draschkow and Vo 2017; Vo 2021). The manipulations can take the form of semantic violations, such as placing objects in the scene that are not typical of the scene category (e.g., a toaster in a playground; Henderson et al. 1999), or syntactic violations of physics (e.g., a toilet on the ceiling; Draschkow and Vo 2017; Vo 2021). Taken together, the evidence suggests that prior knowledge in the form of semantic information facilitates visual search in real-world scenes.

While prior knowledge for scenes clearly informs visual search, there is evidence that recent experience is also a factor in search decisions. Although search for a target in semantically incongruent locations improves with repeated trials, fixations to semantically congruent locations persist (Vo and Wolfe 2012), suggesting that semantic guidance lingers despite repeatedly finding targets in incongruent locations. Young adults appear to be more readily able to learn to search for targets in semantically incongruent locations than older adults (Wynn et al. 2019), which suggests young adults are better able to use both information learned from recent experience and prior knowledge in visual search than their older counterparts, who rely more on prior knowledge. Visual search in abstract displays devoid of scene semantics has been found to be adaptable and sensitive to location probabilities learned from recent experience (Chun and Jiang 1998; Chun 2000; Vickery et al. 2005; Wolfe et al. 2004). Not only do these contextual cueing effects extend to real-world scenes, but observers are able to leverage prior knowledge for scenes to facilitate search when target locations learned from recent experience were predictable (Brockmole and Henderson 2006a, b; Brockmole et al. 2006; Brockmole and Vo 2010). When searching for embedded letter targets (“T” or “L”) in scene images, Brockmole and Vo (2010) found that search became significantly more efficient over time when targets were predictably associated with an object in the scene (the target letter was always on a pillow). The learned association between target letters and pillows generalized to scenes in which the associated object (a pillow) was absent, but could be predicted to occur from the scene context (e.g., a bed with no pillow). However, subjects’ recall for the spatial locations of targets was biased to the spatial center of the associated object rather than the target’s exact location, suggesting that learning of target locations from recent experience was imprecise. While the studies reviewed above suggest that both scene semantics and recent experience inform search decisions, information gained from recent experience may be less reliable, as evidenced by imprecision in spatial memory for target locations (Brockmole and Vo 2010) and the persistence of semantic guidance when search performance would be better served by reliance on recent experience alone (Vo and Wolfe 2012). The current study attempts to determine whether information from prior knowledge and recent experience can be used optimally in visual search.

Search performance in different cognitive domains, such as search in memory, has been shown to be influenced by prior semantic knowledge (Davelaar and Raaijmakers 2012; Anderson 1990; Shiffrin and Steyvers 1997; Duffy et al. 2006; Steyvers et al. 2006; Steyvers and Griffiths 2008; Xu and Griffiths 2010; Hemmer and Steyvers 2009; Hemmer and Persaud 2014; Persaud and Hemmer 2016). For example, Persaud and Hemmer (2016) found that performance in a task measuring color recall was biased by prior knowledge of color categories. Similarly, priors estimated using the subjective beliefs of subjects about a continuous variable (human height) predicted performance in a memory task better than priors constructed from the statistics of the environment (Hemmer et al. 2015). The authors argued that the bias to rely on prior semantic knowledge was the better strategy because semantic knowledge is stable (more reliable) than information gained from recent experience (Steyvers et al. 2006; Hemmer and Steyvers 2009; Hemmer and Persaud 2014). Prior knowledge based on geometry or physical principles can also bias even lower level visuomotor behaviors, such as smooth pursuit eye movements (Santos and Kowler 2017; Badler et al. 2010). The aforementioned studies applied Bayesian models to demonstrate the influence of prior knowledge across a range of behaviors.

Bayesian frameworks provide useful computational tools that can be used to understand how decisions can be determined from prior knowledge combined with immediate evidence (Ma 2012; Beck et al. 2012; Balci et al. 2009; Kheifets and Gallistel 2012; Körding and Wolpert 2004; Todorov 2004; Trommershäuser et al. 2008; Gibson et al. 2013; Traxler 2014; Wang et al. 2018; McCauley et al. 1980; Zaki 2013). One advantage of using Bayesian models is that they can be used to determine possible sources of suboptimal behavior, such as use of an incorrect prior (Ma 2012; Beck et al. 2012), or a failure to encode learned probabilistic information with sufficient fidelity (Dasgupta et al. 2018). Bayesian ideal observer models, which draw optimal inferences conditioned on both prior knowledge and current evidence, have been successfully applied in vision research to characterize object perception (Kersten et al. 2004), contour integration (Feldman 2001), and other perceptual processes (see Geisler 2011 for review). Ideal observer models allow researchers to determine optimal performance on a task using the information available; human performance can then be compared against ideal performance, provided that the relevant stimulus properties can be measured (Geisler 2011). While the performance of the ideal observer on a given task should exceed that of humans, similarity (if not equivalence) between ideal and human performance would suggest that humans make use of the same information in a comparable way to complete the task. In the current study, we expanded on the literature on semantic guidance in visual search by developing a Bayesian ideal observer model with varying dependence on prior knowledge and recent experience. We then compared human performance to that of simulated ideal observers in a visual search task.

The work reviewed above shows that decisions during tasks such as search are strongly influenced by semantic priors and that semantic priors may continue to be influential even when their contribution results in errors. The goal of the current study is to investigate whether searchers can learn to optimally negotiate between using semantic knowledge and recent experience to efficiently locate target objects in a visual search task.

### Rationale of the present study

The present study investigated the trade-offs between prior knowledge and recent experience during an active visual search task that required searching for common objects located within computer-illustrated visual scenes. Search was *active* in that targets were not visible in the scene and thus was carried out by a series of mouse-clicks to reveal the contents of the selected location.

Active search was examined under three conditions: (1) congruent, in which the target location was consistent with the statistical properties of prior knowledge, (2) incongruent, in which that target location was determined by mathematical inversion of the probabilities derived from prior knowledge (i.e., the most probable location under prior knowledge became the least probable location), and therefore, searchers must rely on recent experience to help guide their search, and (3) neutral, in which the target locations were randomly selected from a uniform distribution. To determine whether prior knowledge and recent experience guide search optimally, we developed a Bayesian ideal observer model and simulated performance in the active search task when target locations were learned from the environment perfectly. Agreement between simulated ideal search performance and human search performance would suggest that visual search is Bayesian in that it combines recent experience and prior knowledge optimally to guide search decisions. The active search paradigm allowed us to develop a Bayesian ideal observer model that inferred target locations from prior knowledge and recent experience without the need to model other factors known to influence gaze behavior in visual search (e.g., target size and eccentricity, Schomaker et al. 2017; center bias, Tatler 2007; etc.), or violate scene grammar in the display (Draschkow and Vo 2017).

Two experiments were conducted. In Experiment 1, semantic priors were estimated empirically for targets in two pseudorealistic computer-illustrated scenes. Experiment 1 was conducted to obtain precise estimates of prior expectations about target locations in the main search task, for which experimenter intuition alone would be insufficient (for further discussion, see Chun 2000; Koehler and Eckstein 2017; Henderson and Hayes 2017). Human ratings were used to estimate target location probabilities, which were then used as the basis for target selection in Experiment 2, and constituted prior knowledge in the Bayesian ideal observer model.

Experiment 2 tested search. The same pseudorealistic computer-illustrated scenes shown in Experiment 1 were rendered to be interactive for search in Experiment 2. The target objects appeared in either likely locations that were sampled from a distribution based on prior knowledge (derived from Experiment 1), unlikely locations that were sampled from a distribution that was mathematically derived through the inversion of the prior knowledge (derived from Experiment 1), or random locations. The scenes were searched by mouse click, and points were awarded based on how quickly the target was found. Half of the subjects received feedback about the target’s location at the end of each trial; the remaining subjects received no feedback on the target’s location.

An ideal Bayesian observer was generated to represent the performance that would be obtained when scene semantics and recent experience guide search decisions optimally. Performance in the search task was compared to the performance of Bayesian ideal observers for which search decisions were guided by varying degrees of prior knowledge for scenes, recent experience, or a combination thereof. This allowed us to evaluate how well human searchers were able to learn to prioritize semantic or episodic search guidance in the appropriate context, and how this ability changed over time.

## Experiment 1

In order to measure prior knowledge about target locations in the scenes we designed, Experiment 1 estimated semantic priors for a set of potential targets placed within two scenes (a kitchen and a living room). Subjects rated the likelihood that each potential target would be found in each of the 12 possible locations within the rooms. Ratings were then used to compute the sampling distributions for target locations to be used in Experiment 2 and to model the beliefs of a Bayesian ideal observer.

### Experiment 1: Method

#### Subjects

One-hundred and fifty-three subjects were recruited from Amazon Mechanical Turk. Subjects were screened to have greater than or equal to 1000 approved and completed Mechanical Turk tasks, and had more than 95% of their previous tasks accepted. All subjects were located in the USA. Based on preliminary pilot data, the task was expected to take about 5 min. Subjects were compensated $0.50 per task ($6.00/h).^1^ The study was approved by the Rutgers University Institutional Review Board and procedures were in accordance with the Declaration of Helsinki.

#### Materials

**Fig. 1:**
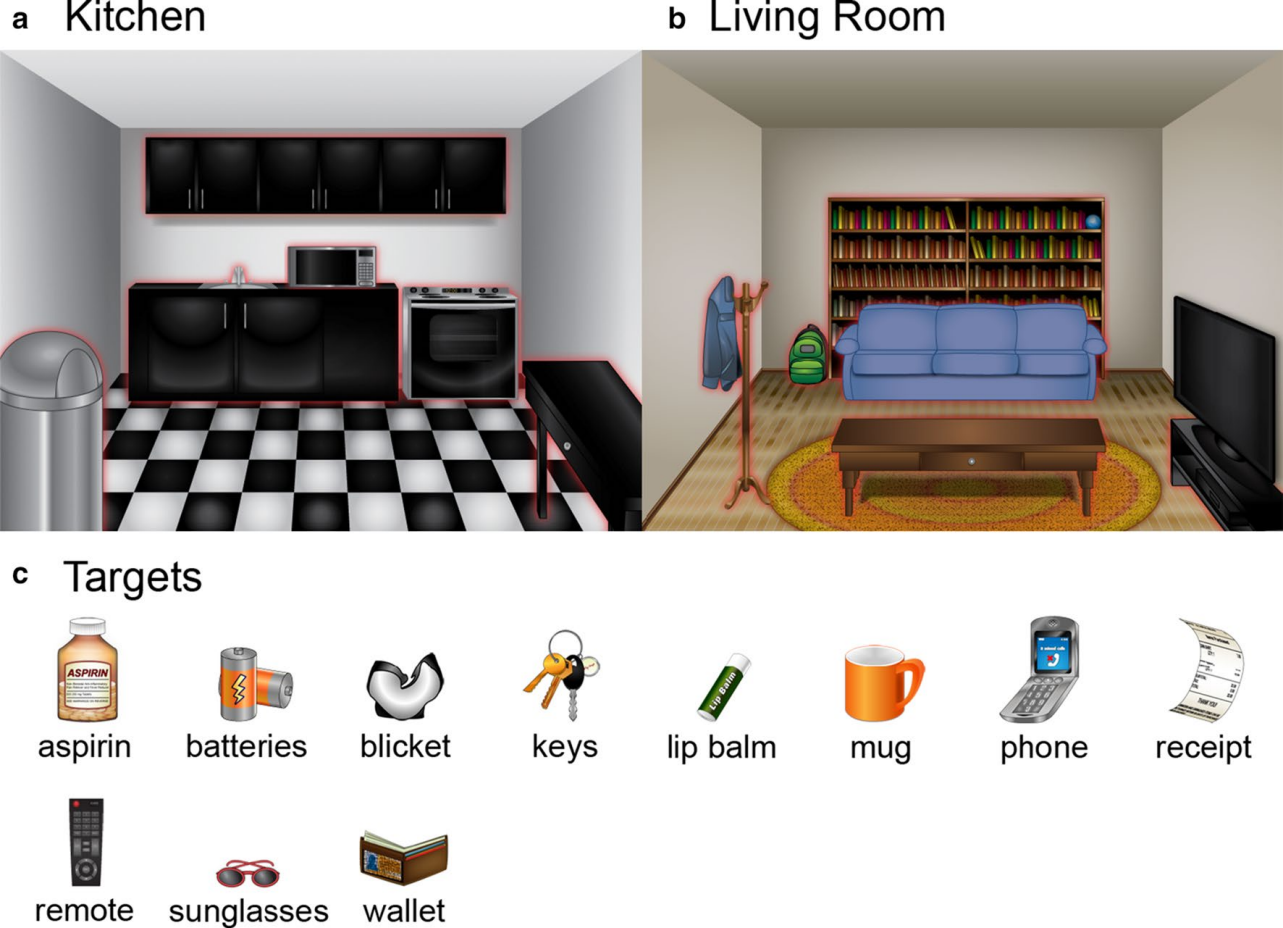
The displays used in Experiments 1 and 2. **a** A kitchen scene including a table, a sink, an oven, a microwave, a trash can, and cabinets. **b** A living room scene including a couch, a table, a television, a bookshelf, a coat, and a backpack. In Experiment 2 only, each searchable location was surrounded by a red glow until the location was searched (as shown in **a** and **b**). **c** 11 targets were rated in Experiment 1: aspirin, batteries, keys, lip balm, a mug, a phone, a receipt, a remote, sunglasses, a wallet, and a novel target: blicket

The experiment was conducted using the Qualtrics Research Suite (Qualtrics, Provo, UT). Drawings of two indoor scenes (800 $\times$ 600 pixels)—a kitchen scene (Fig. 1a) and a living room scene (Fig. 1b)—were displayed side-by-side. Each scene contained 6 potential target locations. Locations in the kitchen were a trash can, a microwave, an oven, a sink, cabinets, a table, and in the living room were a couch, a television, a table, a coat, a bookshelf, and a backpack. We queried eleven potential targets: a remote, a mug, sunglasses, a receipt, a wallet, lip balm, batteries, a phone, keys, aspirin, and a novel target labeled “blicket” (Kouider et al. 2006) (Fig. 1c). Potential targets were common household items that could easily be lost in a room, with the exception of the novel object “blicket”, which served as a validity check on the procedure and analyses in that subjects should not have prior knowledge for its likely or unlikely locations. Target images ranged from 48 to 50 pixels in width and 30–75 pixels in height. Scenes and object images were drawn using Adobe Illustrator vector graphics software.

#### Procedure

For each item, subjects were asked, “Think of the average person. They want to find the [x]. How strongly do you think they will find the [x] in each of the following locations?” (1 = not strongly, 7 = very strongly), where [x] was one of the targets (Fig. 1c). An image of the target was shown below the written prompt, along with images of the scenes as a reference (Fig. 1a, b). Ratings were provided via a 7-point Likert slider, anchored on 1 = not strongly and 7 = very strongly. The slider default was set at 1. For each target, subjects rated all 12 locations separately. Target presentation order was randomized. On average, the survey was completed in 7.70 min ($median = 6.07$ min).

#### Experiment 1: Results

**Fig. 2:**
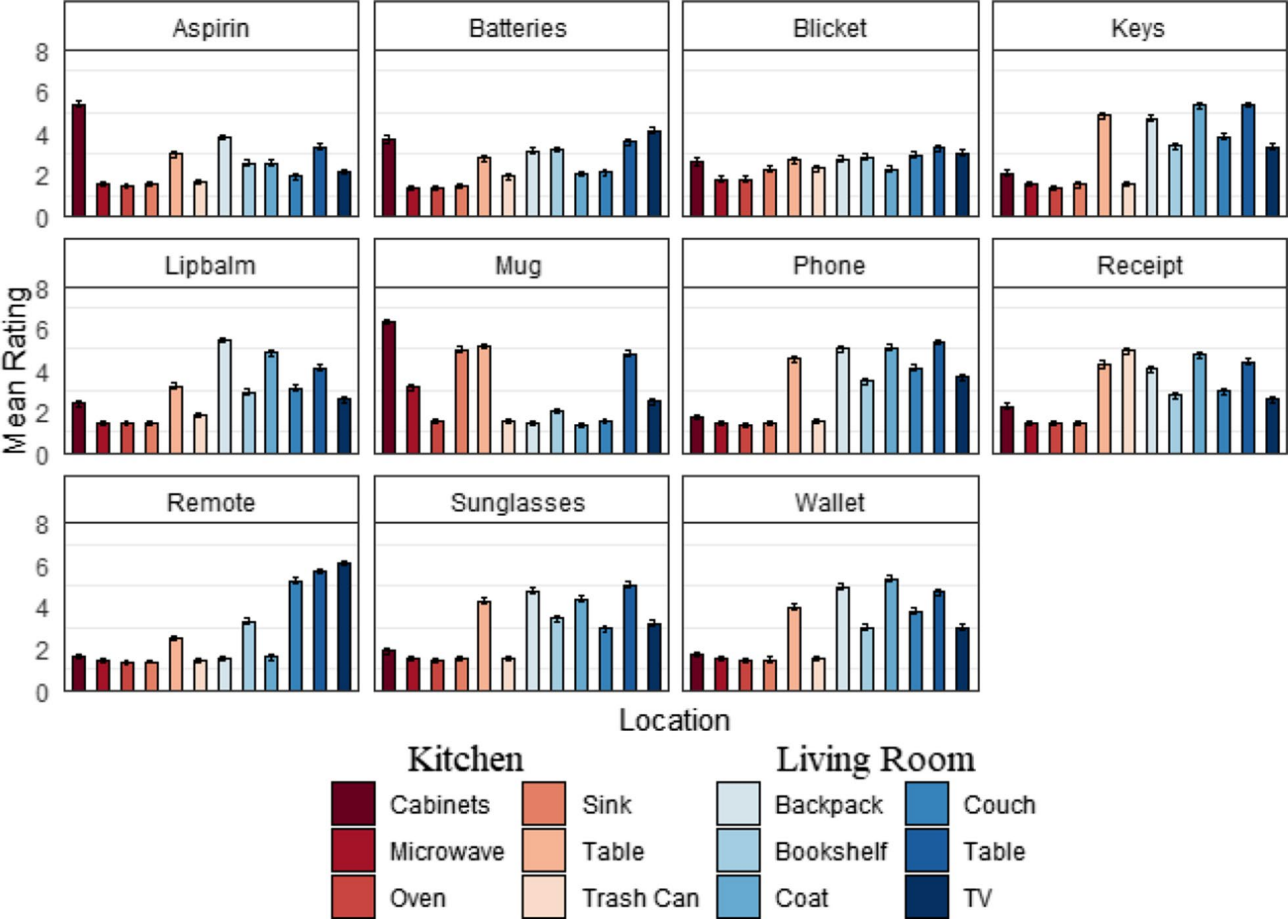
Mean likert ratings for experiment 1. Error bars represent one standard error of the mean ($N =$ 153)

*Likert ratings* Targets were rated more likely to be located in the living room ($M =$ 3.55, SD = 1.21) than in the kitchen ($M =$ 2.25, SD = 1.25). Across targets, the living room table was rated highest on average ($M =$ 4.51, SD = 1.81), and the trash can was rated the lowest ($M =$ 1.97, SD = 1.01). Location ratings depended on the target (see Fig. 2). The mug in the cabinet received the highest average rating ($M =$ 6.28, SD = 1.25), and the phone in the oven received the lowest average rating ($M =$ 1.33, SD = 1.02). Note that for a novel target (the blicket), ratings were approximately uniform.

*Construction of target sampling distributions* To construct the congruent sampling distribution, the probability of each target, *t*, appearing in each queried location, *l*, $\hat{p}_{t,l}$ was estimated from subject’s ratings using multinomial logistic regression:

1 $$\begin{aligned} \hat{p}_{t,l} = {\frac{e^{\bar{L}_t,l}}{\sum _{l'} e^{\bar{L}_t,l'}}} \end{aligned}$$

where ${\bar{L}}_{t,l}$ is the Likert score averaged over the 153 subjects for a target *t* occurring in location *l*. The probabilities obtained using Eq. 1 constituted the sampling distribution for semantically congruent target locations in Experiment 2.

The semantically incongruent target sampling distribution was constructed through mathematical inversion as follows:

2 $$\begin{aligned} \hat{p}_{t,l} = {\frac{e^{-\bar{L}_t,l}}{\sum _{l'} e^{-\bar{L}_t,l'}}} \end{aligned}$$

where the negative of the average Likert score for target *t* occurring in location *l* ($-{\bar{L}}_{t,l}$) was used to invert probabilities. The probabilities obtained from Eq. 2 constituted the sampling distribution for semantically incongruent target locations in Experiment 2.

**Fig. 3:**
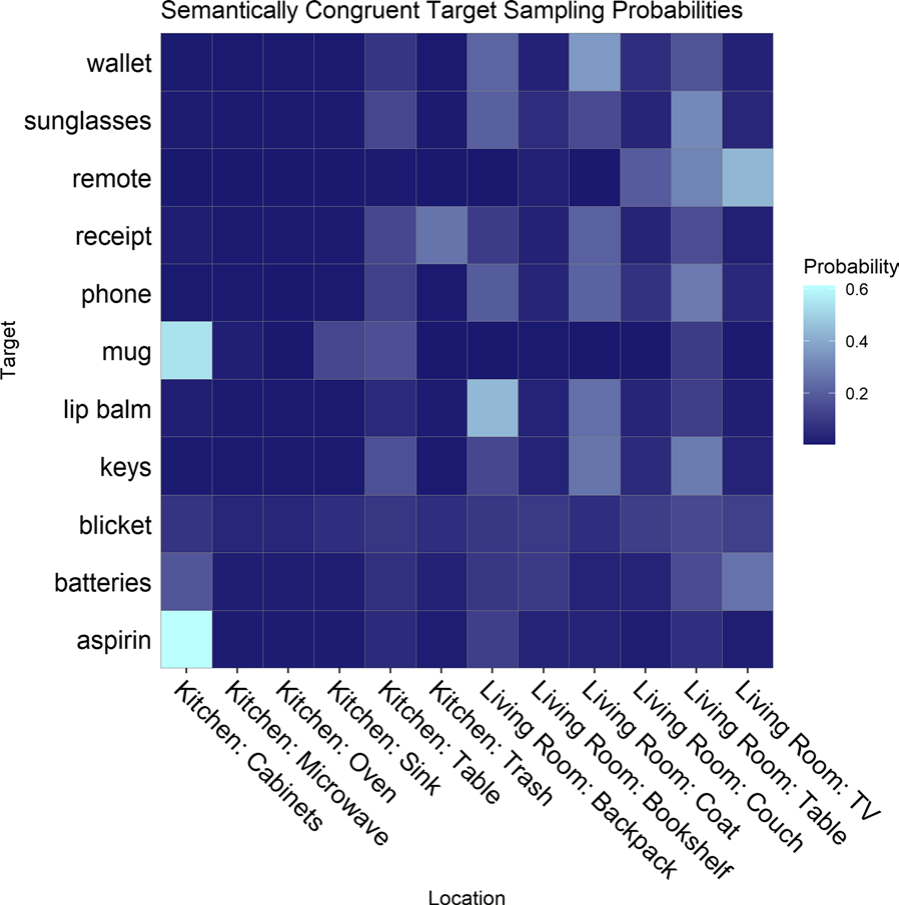
Heatmap displaying the empirically estimated probability of each target (11 targets, *y*-axis) occurring in each location (12 locations, *x*-axis) in the kitchen and living room scenes. The probabilities are congruent with the semantics of the scene

**Fig. 4:**
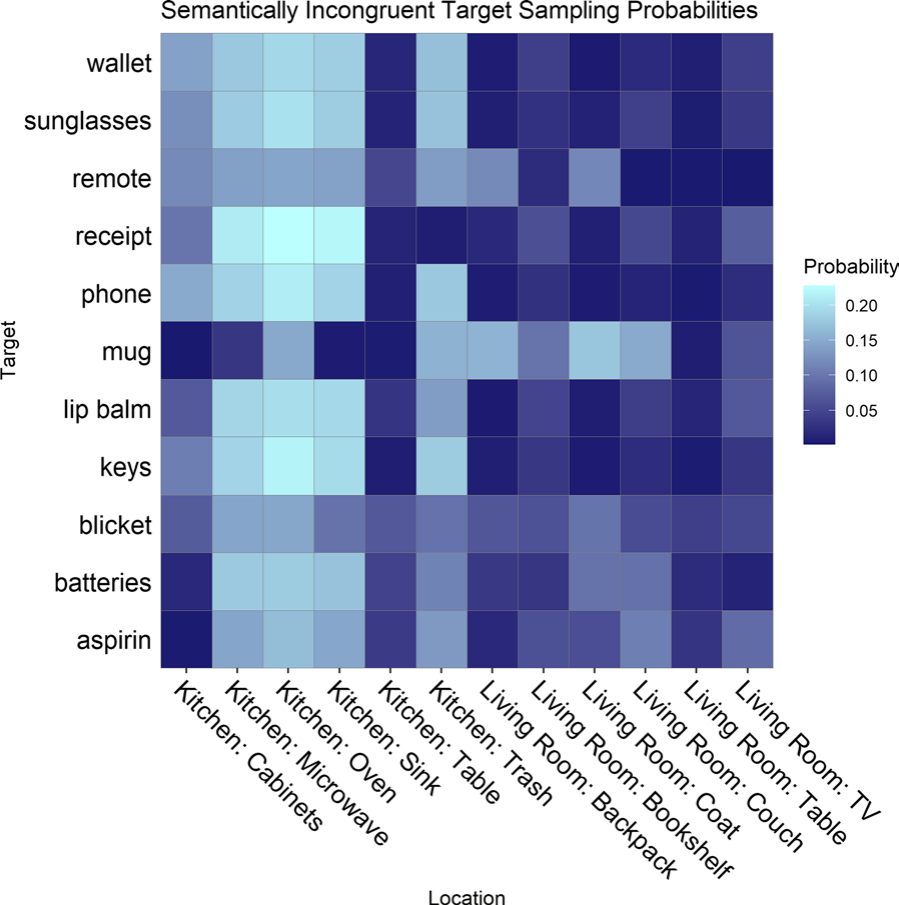
Heatmap displaying the calculated probability of each target (11 targets, *y*-axis) occurring in each location (12 locations, *x*-axis) in the kitchen and living room scenes. The probabilities are incongruent with the semantics of the scene. Note that the scale here differs from the congruent probabilities show in Fig. 3 (the maximum probability value is lower)

*Resulting target sampling distributions* Likert ratings were transformed into probabilities for each target individually (see Figs. 3, 4) using Eqs. 1 and 2. The target-location probabilities served as the basis for selecting targets to use in the search task and constituted the sampling distribution for the selected targets.

### Experiment 1: Discussion

Experiment 1 estimated the prior beliefs about the probability that a given location would contain the target. We found that all targets (except for the novel “blicket”) showed non-uniform expectancies of likely locations. The distributions were not symmetric in that the semantically incongruent distribution (Fig. 4) was flatter than the semantically congruent distribution (Fig. 3).

Two targets had a strong bias for one location (the mug and the aspirin in the cabinets) while the other targets, with the exception of the novel target, were biased toward multiple locations. Only three of the 11 targets were chosen to examine learning for each target under different congruency conditions over several trials, without subjects becoming fatigued. The three targets were selected for the search task (Experiment 2) based on their location probabilities: the mug, batteries, and keys. The keys and the batteries were selected because they were rated as likely to be found in different locations (e.g., keys in the coat or on the table, batteries in the television; see Fig. 3), and were rated as unlikely to be found in the same locations (e.g., neither keys or batteries were likely to be found in the oven or microwave; see Fig. 4). The mug was chosen because it was rated as highly likely to be found in a single location (the cabinet, see Fig. 3), which suggests that prior expectations for the location of mugs were strongly biased toward that location. We limited the number of targets to these three in order to compare search performance for targets with dissimilar sampling distributions with enough observations to do so effectively.

These measures directly quantify prior knowledge for target locations in the stimuli, and thereby allowed us to implement a Bayesian ideal observer model for comparison against human search performance.

## Experiment 2

The goal of the search task (Experiment 2) was to determine whether semantic and episodic information (recent experience with target locations) optimally guide search decisions. Target locations were selected to be either (1) congruent with scene semantics, from the congruent sampling distribution derived in Experiment 1 (semantically congruent), (2) incongruent with scene semantics, from the incongruent sampling distribution computed in Experiment 1 (semantically incongruent), or (3) random. We conducted a power analysis through G*Power 3.1.9.2 (Faul et al. 2007) to determine our sample size. With an alpha of .05, the power analysis revealed that a sample of 10 subjects would allow us to achieve a projected power of .95 and a projected effect size of .25 for a three-way repeated measures analysis of variance to compare subjects’ search performance on the factors: target (three levels), congruence (three levels), and session number (five levels).

### Experiment 2: Method

#### Subjects

Ten subjects completed the experiment. Data from one additional subject was collected but not analyzed because the instructions were misinterpreted. All subjects had normal or corrected-to-normal vision. Subjects were paid $10/h. The study was approved by the Rutgers University Institutional Review Board and was conducted in accordance with the Declaration of Helsinki.

#### Apparatus

Data was collected on a Dell Optiplex 755 with a 21.5” Dell SX2210Tb monitor (60 Hz refresh rate) using 1920 $\times$ 1080 desktop resolution. The experiment was written in HTML, CSS, and JavaScript using jsPsych (de Leeuw 2015), KineticJS version 5.1.0 (Rowell et al. 2012), and jQuery version 1.11.1 (jQuery Foundation, Inc.). The experiment was presented in a maximized Google Chrome browser window. Viewing distance was whatever felt most comfortable for the subject, which ranged from approximately 20-24”.

#### Stimuli

**Fig. 5:**
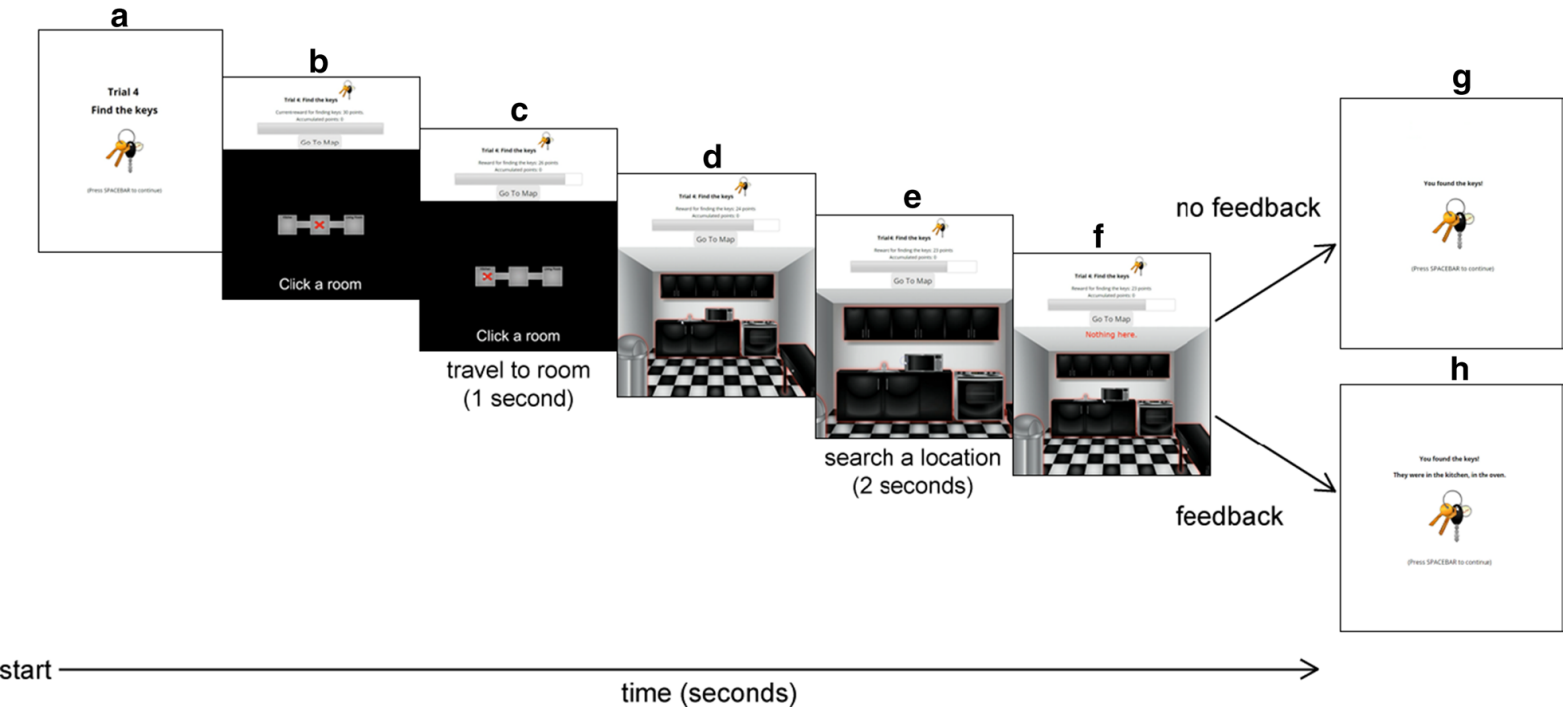
Schematic of the trial procedure

Stimuli for the search task were the same two room scenes used in Experiment 1 (Fig. 1a, b). Three displays were constructed using Adobe Illustrator and Adobe Photoshop, and were rendered as interactive scenes for the search task: a kitchen scene (Fig. 1a), a living room scene (Fig. 1b), and a map (Fig. 5b, c). Six searchable locations in each room were surrounded by a red glow (which can be seen in 1a, b) until searched. An image of the target (48-75 pixels wide and 48-93 pixels high) appeared on each trial above the display and alongside the task instructions. A simple “map” consisted of an 800 $\times$ 600 drawing with three grey, equally spaced squares (87 $\times$ 84), a red “ $\times$ ” (82 $\times$ 84) to mark the current location on the map, and the text “Click a room”. The outermost squares on the map were labeled with room names (right: “Kitchen”, left: “Living Room”) and could be used to switch rooms via mouse click.

The display screen additionally showed the trial number, the subject’s current trial score (e.g., “Current reward for finding the [target]: 22 points”), the cumulative score for the block (“Accumulated points”), a button to access the map, the 800 $\times$ 600 region where the map and scenes were displayed, and a small image of the target. A 500 pixel-wide timer bar indicated the time (and possible points) remaining in the trial. The timer bar and the maximum possible score were updated every second to reflect the time remaining in the trial. Below the score, a grey button labeled “Go To Map” allowed subjects to access the map.

#### Design

The levels of semantic congruence were (1) *Semantically congruent*: target probabilities were congruent with the semantics of the scene, selected using the congruent sampling distribution obtained in Experiment 1 (see Eq. 1), (2) *random*: target location probabilities were random; and (3) *semantically incongruent*: target location probabilities were selected using the incongruent sampling distribution derived from the data in Experiment 1 (Eq. 2), and thus target locations were incongruent with the scene semantics. The congruence manipulation was implemented via experimental blocks. In each block, rooms were searched for three targets: a mug, batteries, and keys (Fig. 1c). There was one search target per trial (10 trials/target/block).

#### Procedure

*Order of testing* Each experimental session (approximately 45 min) consisted of three blocks of 30 trials (10 trials per target), one block for each level of semantic congruence. Subjects were tested for 5 sessions, except for one subject who was tested for only 4 sessions. Each session took place on a separate day. The order of blocks was pseudorandomized such that no two subjects received the same congruence condition order across sessions, and no subject received the same block order across sessions. Within a block, targets were selected at random. Each of the three targets appeared 10 times without replacement in a 30 trial block.

*Instructions* Before the beginning of testing, subjects were told that they would be searching two computer-illustrated rooms for a target. They were given a list of searchable locations within each room. Subjects were informed that they would earn points by finding the target, and that they would earn more points for finding the target quickly.

*Familiarization* Each block was preceded by 12 familiarization trials to inform subjects about the searchable locations within each of the two scenes. In the familiarization trials, each room scene was displayed one at a time, in randomized order. In each scene, the six searchable locations were outlined with a red glow. Subjects were provided a label for one of the locations and instructed to click on it (e.g., “Click on the microwave”). Upon clicking a location, a sound played (“cha-ching” if they correctly clicked on the instructed location or “splat” for an incorrect click). Feedback text in 30 pt red font (“Correct!” or “Incorrect!”) was overlaid on the top-center of the scene for 900 ms. The trial persisted until the correct location was clicked, at which point there was a 250 ms inter-trial interval and subsequently the next familiarization trial was displayed. Familiarization trial order was randomized without replacement. Once all six locations in the first scene were correctly identified, the same procedure was repeated for the second scene.

*Search task* Before each experimental block, a fictitious street address with a randomly generated house number was displayed and subjects were asked to search for items within the house. The purpose of the address was to produce the impression that subjects were searching in a new house in each block of trials. The address persisted until the subject pressed a key to begin the block.

A trial proceeded as follows (see Fig. 5). First, a screen displayed the current trial number and instructed the subject to find the displayed target (Fig. 5a). This screen remained until the space bar was pressed, which started the trial. A timer recorded the duration of the trial.

To initiate search, subjects first used the map to select a room (Fig. 5b), which caused the symbol (x) on the map to be displaced to the center of the chosen room over a period of 1000 ms (Fig. 5c). Subjects were able to switch between rooms at any time by clicking on a “Go to Map” button above the display region. Within a room, subjects could click on one of the searchable locations designated by the red glow (Fig. 5d). Upon clicking a location to search, an animation briefly (2000 ms) zoomed in on the chosen location (150% scale) and then zoomed out to the full scale of the room (Fig. 5e), during which time subjects were unable to act. Following the search animation, audio and text feedback about the outcome of the search were provided simultaneously (Fig. 5f): auditory feedback was the same as in the familiarization task, and text feedback was overlaid in 30 pt red font (“Nothing here.” or “Found!”) for 900 ms. After searching a location, the red glow surrounding the location disappeared, and subjects could no longer interact with the location. If the target was in the chosen location, a “cha-ching” sound played, the message “Found!” (900 ms) was displayed, and the trial terminated. A screen then appeared indicating that the target was found, and displayed an image of the target (Fig. 5g). Subjects then moved to the next trial. Otherwise, a “splat” sound played, and the message “Nothing here” (900 ms) was displayed, after which they could continue searching. The trial persisted until either the target was found or 30 seconds elapsed. If the target was not found within 30 seconds, a screen appeared indicating that they did not find the target, along with an image of the target (Fig. 5h).

*Point system* To measure search performance, points were awarded equal to the seconds remaining in the trial at the time that the target was found. Delays associated with selecting a room to search in (1000 ms) and searching a location within a room (2000 ms) cost subjects 1 and 2 points, respectively. This meant that a maximum of 27 points could be earned in a trial if the target was found immediately. Points awarded were the inverse of reaction time (e.g., 27 points earned corresponded to a 3 second search). If the target was not found, zero points were awarded. Points awarded on each trial were added to a cumulative score over the course of a block as motivation for subjects, but did not carry over to subsequent blocks.

*Feedback* Half of the subjects received feedback regarding the actual location of the target at the end of a trial whether the target was found during the trial or not (Fig. 5h). The other 5 subjects were not informed of the target’s actual location after each trial (Fig. 5g).

#### Analysis

There were a total of 450 trials per subject (10 trials per target $\times$ 3 targets per block $\times$ 3 blocks per session $\times$ 5 sessions per subject) for 9 subjects (4050 trials), and 360 trials for one subject who completed only 4 experimental sessions, yielding a total of 4410 trials across subjects. Sixteen trials were excluded from analysis due to a browser rendering issue during data collection. Data from the remaining 4394 trials were analyzed.

*Analysis of search performance* Search performance was measured in two ways: points earned and reaction time. Both measures were analyzed using a mixed analysis of variance. There were three within-subjects factors: (1) the semantic congruence of the environment (semantically congruent, random, or semantically incongruent), (2) the experimental session number (1–5) to evaluate learning, and (3) the target (mug, batteries, or keys). Whether or not the subject received feedback at the end of each trial was included as a between-subjects factor.

### Experiment 2: Ideal observer model and simulations

We developed a Bayesian model to predict the behavior of an ideal observer conditioned on prior knowledge and recent experience.

### Ideal observer model

An ideal observer’s belief that a target *t* would be found in a location *l* during search *i*, based on the searcher’s prior knowledge *w* and recent experience *r*, can be expressed using Bayes rule as follows:

3 $$\begin{aligned} P(\theta _{t,l}|r,w) = \frac{ P(r|\theta _{t,l})P(\theta _{t,l}|w)}{\sum _{i,j}P(r|\theta _{t_{i},l_{j}})P(\theta _{t_{i},l_{j}}|w)} \end{aligned}$$

where $\theta _{t,l}$ represents the searcher’s belief that target *t* is in location *l*, *r* represents the searcher’s recent experience, and *w* represents the searcher’s prior expectations based on general world knowledge. Note that before the first search event takes place, $\theta _{t,l}$ is determined by *w*.

To model an ideal observer’s beliefs, we treated each search event as a Bernoulli trial, where the outcome is either a success or a failure. Prior beliefs derived from knowledge about scenes were represented by a Beta distribution because it is well-suited to represent binary-event probabilities (Kruschke 2014). We estimated the expected value, $\hat{p}_{t,l}$, of the Beta distribution representing prior knowledge about scene semantics using the Likert ratings obtained in Experiment 1 as follows:

4 $$\begin{aligned} \hat{p}_{t,l} = \frac{\bar{L}_{t,l}}{7} \end{aligned}$$

where ${\bar{L}}_{t,l}$ is the Likert score averaged over raters for a target *t* occurring in location *l*, and 7 is the maximum value on the Likert scale. To obtain a probability estimate, we divided ${\bar{L}}_{t,l}$ by 7, the maximum value of the Likert scale.

We obtained the Beta distribution shape parameter $\alpha _{t,l}$, the observed number of successful searches for the target *t* in location *l*, and the scale parameter $\beta _{t,l}$, the number of times the target *t* was not found when location *l* was searched, by multiplying the expected value of each prior distribution ($\hat{p}_{t,l}$) by the total number of observations, *s*, as follows (Eq. 5)

5 $$\begin{aligned} P(\theta _{t,l}|r,w) = \Pi _i P(t_{f}^{i}|\theta _{t,l})P(\theta _{t,l}|\alpha _{t,l},\beta _{t,l}) = \Pi _i P(t_{f}^{i}|\theta _{t,l})P(\theta _{t,l}|\hat{p}_{t,l},s) \end{aligned}$$

where $\theta _{t,l}$ represents the event that the target is found (Ferrari and Cribari-Neto 2004). Because *s* is the total number of observations, it determines how heavily the prior $\hat{p}_{t,l}$ influences search behavior, and therefore *s* determines whether the searcher relies more on prior knowledge or on information gained from recent experience. We refer to *s* in the simulations as prior strength.

#### Simulations

We used the ideal observer model to predict optimal search performance for the target objects. The simulation (1) determined the behavior of an ideal observer for the task, and (2) predicted behavior as a function of different levels of the searcher’s dependence on world knowledge or recent experience (see appendix for expanded simulation methods).

Three levels of semantic congruence were tested: (1) *Semantically congruent*: target probabilities were congruent with the semantics of the scene, selected using the congruent sampling distribution obtained in Experiment 1 (see Eq. 1), (2) *random*: target location probabilities were random; and (3) *semantically incongruent*: target location probabilities were selected using the incongruent sampling distribution derived from the data in Experiment 1 (Eq. 2). The three levels of semantic congruence, the sampling distributions for the three targets (as found in Experiment 1), and the target location sampling method were all identical to those used in the search task.

To compare performance of the simulated searcher against humans who have physical limitations (e.g., the need to move a mouse), costs associated with search were doubled: selecting a room to search deducted the simulated searcher’s score by 2 points (vs. 1 for humans), and searching a location resulted in a 4 point deduction (vs. 2 for humans). Six different ideal observers were tested, varying in their dependence on prior knowledge. The dependence of the simulated searcher’s beliefs on prior knowledge was termed *prior strength*. Low values for prior strength simulated a strong reliance on recent experience, while high values simulated a strong reliance on prior beliefs.

**Fig. 6:**
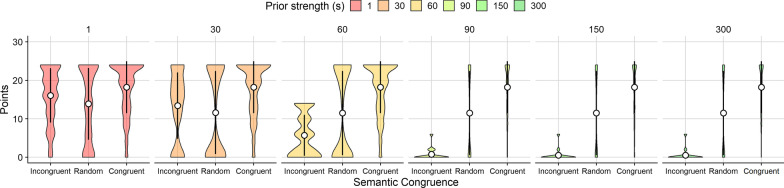
Violin plots showing showing points earned by the ideal observer for different levels of prior strength (columns), as a function of semantic congruence. Low values for prior strength simulated a strong reliance on recent experience, while high values simulated a strong reliance on prior beliefs. Each violin plot corresponds to *N* = 1500 simulated trials. White circles indicate the mean and error bars represent ±1 standard deviation

Simulation performance was assessed via the points earned in the semantically incongruent condition, in which targets were placed in the least likely locations under prior knowledge guided by scene semantics. We simulated 500 trials for each of the three targets in each congruence condition (semantically congruent, random, semantically incongruent), and each prior strength value (1, 30, 60, 90, 150, 300) resulting in 27,000 total simulated trials. Results of the simulation showed that simulated searchers successfully prioritized information from recent experience over prior knowledge when prior strength was below 60 (Fig. 6).

**Fig. 7:**
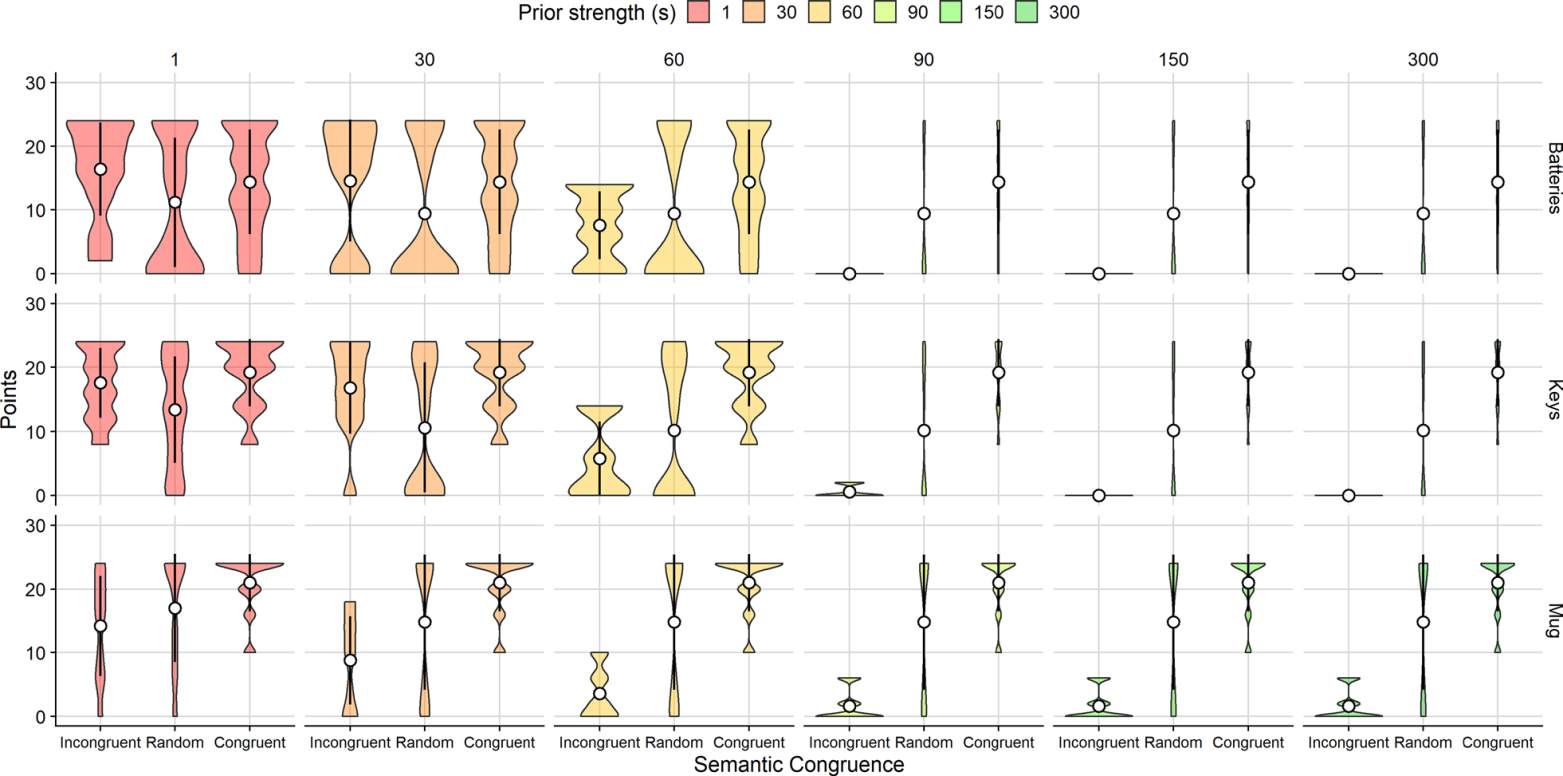
Violin plots showing points earned by the ideal observer for 3 targets (rows) for different levels of prior strength (columns), as a function of semantic congruence. Low values for prior strength simulated a strong reliance on recent experience, while high values simulated a strong reliance on prior beliefs. Each violin plot corresponds to *N* = 500 simulated trials. White circles indicate the mean and error bars represent $\pm 1$ standard deviation

Considering performance for each target separately, simulated searchers learned to search successfully for all targets except the mug (Fig. 7), suggesting preliminarily that statistical learning of the incongruent target locations for the mug was not possible.

These simulations predicted the ideal search performance for the three targets chosen for the active search task under varying degrees of reliance on world knowledge and recent experience, as determined by prior strength (*s*). Because the ideal observer performed similarly for prior strength values over 60 when target locations were incongruent with scene semantics (per Fig. 6), we chose to compare human performance in the search task to the simulated searcher’s performance using prior strength values of 1 (driven by recent experience), 60 (informed by both prior knowledge and recent experience), and 300 (driven by world knowledge).

### Experiment 2: Results

#### Search performance

**Fig. 8:**
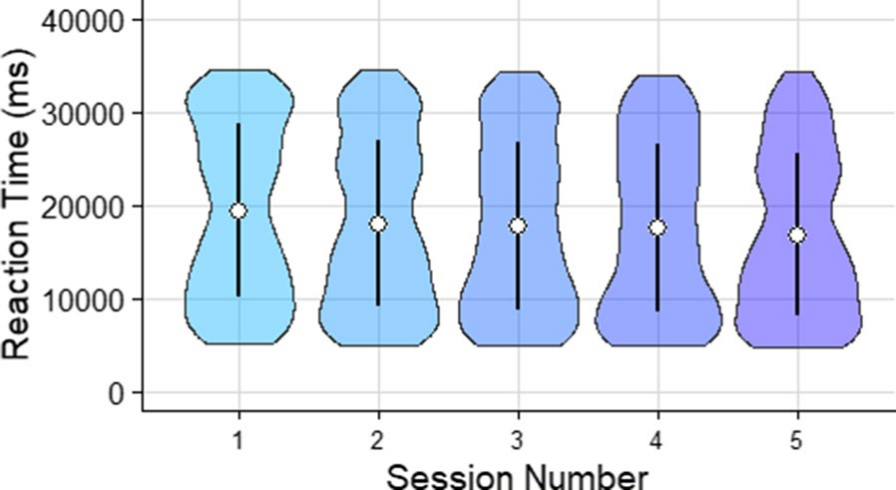
Violin plot illustrating points earned per experimental testing session ($N_{Session_1} =$ 892, $N_{Session_2} =$ 900, $N_{Session~3} =$ 898, $N_{Session_4} =$ 895, $N_{Session_5} =$ 809). White circles indicate the mean. Error bars show $\pm 1$ standard deviation

Mean reaction time was analyzed using a mixed ANOVA with repeated measures on semantic congruence, session, and target and feedback as a between subjects measure. There was no effect of feedback, *F*(1,7) $=$ 1.863, $p =$ .215, $\eta ^{2} =$ .210, on participants’ reaction time and so data was collapsed across feedback conditions (Fig. 8).

**Fig. 9:**
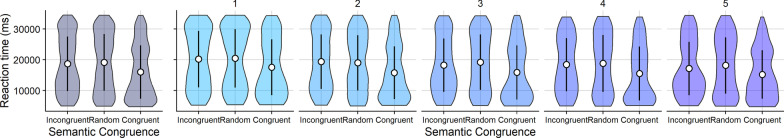
Violin plots indicating reaction time in each semantic congruence condition (left, $N_{Incongruent} =$ 1467, $N_{Random} =$ 1464, $N_{Congruent} =$ 1463), and additionally for each experimental session (right, each violin plot is based on between 269 and 300 observations). White circles represent the mean and error bars represent $\pm 1$ standard deviation

There were main effects of session, *F*(4,32) $=$ 5.769, $p =$ .001, $\eta ^{2} =$ .419, and congruence, *F*(2, 16) $=$ 17.665, $p<$ .001, $\eta ^{2} =$ .688. Pairwise comparisons with Bonferroni corrections indicate that mean reaction time from session 1 was significantly different from sessions 2 and 5. Specifically, reaction time in session 1 was significantly slower than sessions 2, $p =$ .011, and 5, $p =$ .005. All other comparisons were insignificant. For semantic congruence, participants were quicker in the semantically congruent condition than the other two congruency conditions, $ps<$ .006 (Fig. 9).

**Fig. 10:**
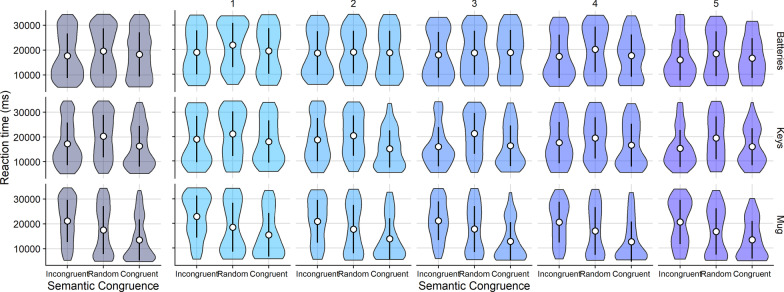
Violin plots indicating reaction time for each target in each semantic congruence condition (left, each violin plot is based on between 487 and 490 observations), and additionally for each session (right, each violin plot is based on between 89 and 100 observations). White circles represent the mean and error bars represent $\pm 1$ standard deviation

A significant interaction between congruence and target was also found, *F*(4,32) $=$ 22.391, $p<$ .001, $\eta ^{2} =$ .737. Reaction time was higher when participants were searching for the mug in the semantically incongruent condition ($M =$ 20791.50 ms, $SE =$ 672.67 ms) than the batteries ($M =$ 17384.59 ms, $SE =$ 537.73 ms) and the keys ($M =$ 16724.22 ms, $SE =$ 812.18 ms), but lower in the semantically congruent condition ($M =$ 13396.55 ms, $SE =$ 967.58 ms) in comparison to the batteries ($M =$ 17821.19 ms, $SE =$ 661.62 ms) and the keys ($M =$ 16359.11 ms, $SE =$ 917.67 ms). There were no other significant interactions (Fig. 10).

**Fig. 11:**
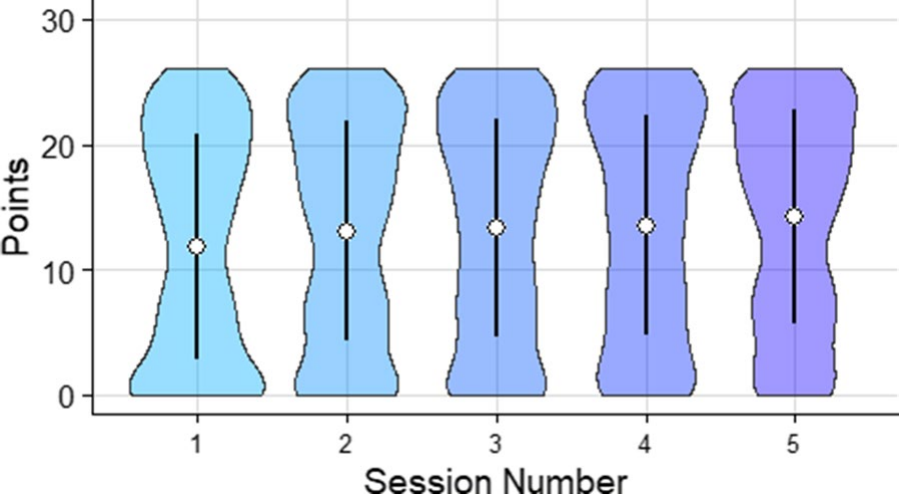
Violin plot illustrating points earned per experimental testing session ($N_{Session_1} =$ 892, $N_{Session_2} =$ 900, $N_{Session~3} =$ 898, $N_{Session_4} =$ 895, $N_{Session_5} =$ 809). White circles indicate the mean and error bars represent ±1 standard deviation

Points earned in each trial was used to compare the performance of human searchers to that of the ideal observer. The average points per subject for all trials in each block were analyzed using a mixed ANOVA with repeated measures on semantic congruence, session, and target and feedback as a between subjects measure. The ANOVA revealed no effect of feedback *F*(1, 7) $=$ 2.067, $p =$ .194, $\eta ^{2} =$ .023, therefore, feedback was not included as a factor in subsequent analyses (Fig. 11).

**Fig. 12:**
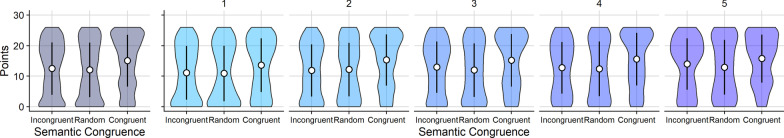
Violin plots indicating points earned in each semantic congruence condition (left, $N_{Incongruent} =$ 1467, $N_{Random} =$ 1464, $N_{Congruent} =$ 1463), and additionally for each experimental session (right, each violin plot is based on between 269 and 300 observations). White circles represent the mean and error bars represent $\pm 1$ standard deviation

A three-way repeated measures ANOVA revealed a main effect of session, *F*(4,32) $=$ 5.528, $p =$ .008, $\eta ^{2}$ = .409, and congruence level, *F*(2,16) $=$ 18.197, $p =$ .001, $\eta ^{2} =$ .695. On average, scores increased over sessions (Fig. 7). Pairwise comparison using Bonferroni correction revealed significantly higher scores by the fifth session relative to the first, $p =$ .003, and higher scores by the second session relative to the first, $p =$ .014. Other pairwise contrasts were not significant (Fig. 12).

**Fig. 13:**
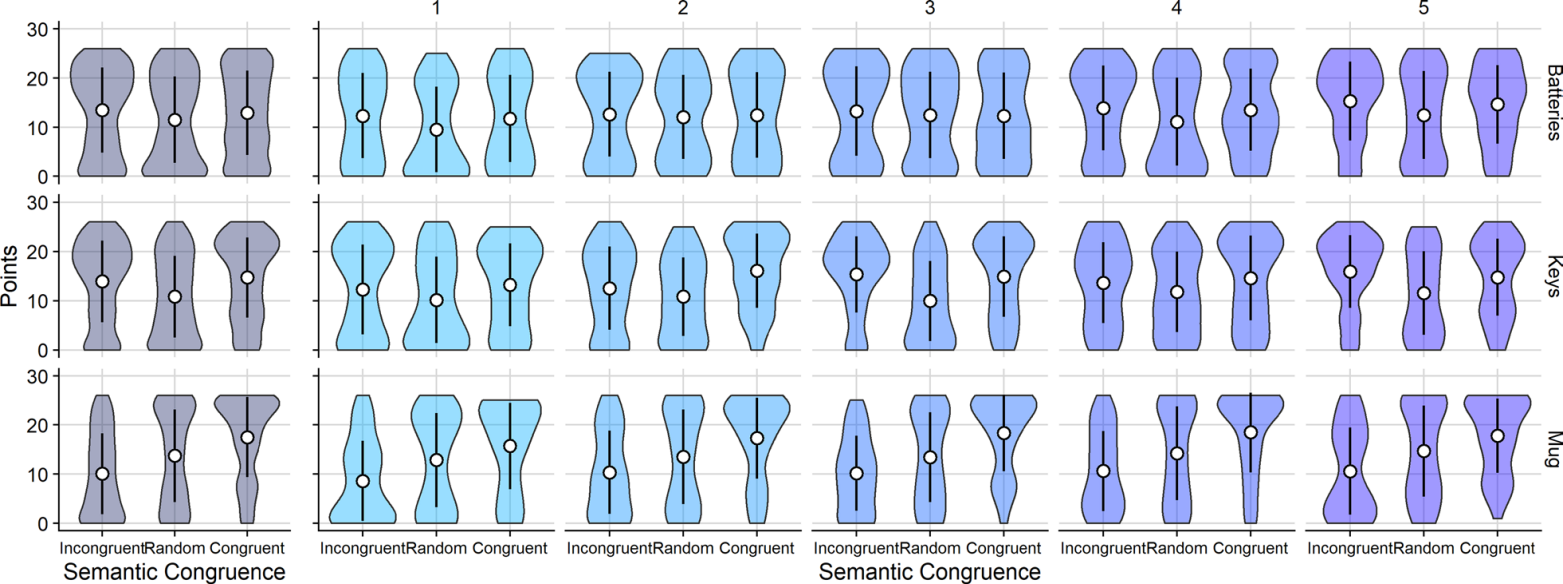
Violin plots indicating points earned for each target in each semantic congruence condition (left, each violin plot is based on between 487 and 490 observations), and additionally for each session (right, each violin plot is based on between 89 and 100 observations). White circles represent the mean and error bars represent $\pm 1$ standard deviation

Scores were higher in the semantically congruent condition than in both the incongruent, $p =$ .002, and the random condition, $p =$ .006; (Fig. 12). There was no difference between the semantically incongruent condition and random condition ($p =$ .40). There was no main effect of target on overall points accrued, *F*(2,16) $=$ 2.471, $p =$ .134, $\eta ^{2} =$ .236 (Fig. 13).

There was a significant interaction between target and congruence level, *F*(4,32) $=$ 22.596, $p<$ .01, $\eta ^{2} =$ .739, suggesting that there was a significant effect of the target on the ability to learn incongruent target locations as predicted by the simulation. Namely, searchers would be less able to learn to search successfully in the semantically incongruent condition when searching for the mug than for the other targets, given that the ideal observer was unable to learn the mug’s incongruent locations. The results for human searchers supported this prediction (see Fig. 12). When searching for the mug, scores were highest in the semantically congruent condition ($M_{\mathrm{{mug}}} =$ 17.659, $\mathrm{{SE}}_{\mathrm{{mug}}} =$ .943, $N_{\mathrm{{mug}}} =$ 393) and lowest in the incongruent condition ($M_{\mathrm{{mug}}} =$ 10.416, $\mathrm{{SE}}_{\mathrm{{mug}}} =$ .623, $N_{\mathrm{{mug}}} =$ 396), indicating difficulty when learning to search for the mug. In contrast, learning to search for the batteries or the keys was tractable. Scores for the batteries and keys were similar in the semantically congruent ($M_{\mathrm{{batteries}}} =$ 13.339, $\mathrm{{SE}}_{\mathrm{{batteries}}} =$ .630, $N_{\mathrm{{batteries}}} =$ 418; $M_{\mathrm{{keys}}} =$ 14.637, $\mathrm{{SE}}_{\mathrm{{keys}}} =$ .950, $N_{\mathrm{{keys}}} =$ 408) and incongruent condition ($M_{\mathrm{{batteries}}}$ = 13.772, $\mathrm{{SE}}_{\mathrm{{batteries}}} =$ .526, $N_{\mathrm{{batteries}}} =$ 416; $M_{\mathrm{{keys}}} =$ 14.436, $\mathrm{{SE}}_{\mathrm{{keys}}} =$ .776, $N_{\mathrm{{keys}}} =$ 410), and lowest in the random condition ($M_{\mathrm{{batteries}}} =$ 11.634, $SE_{\mathrm{{batteries}}} =$ .614, $N_{\mathrm{{batteries}}} =$ 404; $M_{\mathrm{{keys}}} =$ 11.034, $SE_{\mathrm{{keys}}} =$ .585, $N_{\mathrm{{keys}}} =$ 401; see Fig.  13). There was no interaction between session number and either the congruence level, *F*(8,64) $=$ .244, $p =$ .98, $\eta ^{2} =$ .030, or the target, *F*(8,64) $=$ .364, $p =$ .814, $\eta ^{2} =$ .044.

Because points decreased as time elapsed in a trial, reaction time and points earned were strongly correlated with one another (Pearson’s $r(4392) = -0.99, p<$ .0001). To compare subjects’ search performance with the simulated ideal observer, for which response times were not available, we elected to compare points earned by human searchers to that of the simulated searchers under three levels of prior strength that predicted different search performance in the simulations: 1, 60, and 300.

#### Comparison to simulations

To explore how well humans searched by comparing their performance to the simulations, we compared points earned by the human searchers in each experimental block with the ideal observer’s performance at prior strength values of 1, 60, and 300 (selected based on Fig. 6). Experimental blocks in which target locations were random were excluded from analysis in order to limit the number of comparisons to those that would be most informative and interpretable. We conducted two-tailed unpaired Bayesian *t*-tests using the ‘BayesFactor‘ package in R (Morey and Rouder 2011) to compute Bayes factors that weigh evidence for the null ($H_{0}$: no difference between sample means) against the alternative hypothesis ($H_{1}$: sample means are different; Rouder et al. 2009). Evidence in favor of the null ($BF_{01}$) was calculated by inverting the default Bayes factors ($BF_{10}$) that assess evidence for the alternative hypothesis against the null (($\frac{H_{1}}{H_{0}}$ )$^{-1} =$ $\frac{H_{0}}{H_{1}}$ )—in this case, $BF_{10}$ captures similarity to the ideal observer. For the current analysis, Bayes factors above 1 were considered to support the null hypothesis, and the magnitude of the ratio indicated the strength of the evidence.

For each subject ($\hbox {n} = 10$), each congruence block ($\hbox {n} = 2$) in each session ($\hbox {n} = 5$ for all subjects but one) was compared to aggregated data from the simulations. Because there were 1500 simulated trials for each level of prior strength and congruence condition, simulated data were averaged over bins of 50 trials, resulting in 30 data points (means) for each level of prior strength (1, 60, and 300) and semantic congruence condition (congruent or incongruent) for comparison (n = 6 samples). There were 588 *t*-tests in total. The number of tests that favor the null in each condition are reported.^2^

Aggregated performance for ideal observers in the congruent condition was approximately identical for all levels of prior strength, therefore for the congruent condition, we report only comparisons to the performance of the simulated searcher with the lowest prior strength ($s =$ 1).

*Congruent human performance.* For overall subject performance, a total of 23 Bayes factors (47%) exceeded 1, and the average Bayes factor was 2.72 (SD = 0.86), suggesting the overall performance for approximately half of subjects was comparable to the ideal observers that relied on recent experience exclusively ($s =$ 1) in the congruent condition. However, the number of Bayes factors that exceeded 1 was higher ($n =$ 34, 69%) when human data from the congruent condition was compared to the ideal observer relying only on recent experience ($s =$ 1) in the incongruent condition, and the average Bayes factor was slightly higher ($M =$ 2.80, SD = 0.84), suggesting human subjects searching in the congruent condition performed slightly worse than the simulated searcher that relied on recent experience in the congruent condition, and were more similar to the ideal observer that relied on recent experience in the incongruent condition. In contrast, only 2 Bayes factors supported the null when subject data was compared performance of the ideal observer with partial world knowledge ($s =$ 60) in the incongruent condition ($M =$ 1.80, SD = 0.99), in sessions 1 ($BF_{01} =$ 2.50) and 4 ($BF_{01} =$ 1.10), and 0 Bayes factors supported the null when subject data was compared to simulated performance that relied solely on world knowledge in the incongruent condition ($s =$ 300).

**Fig. 14:**
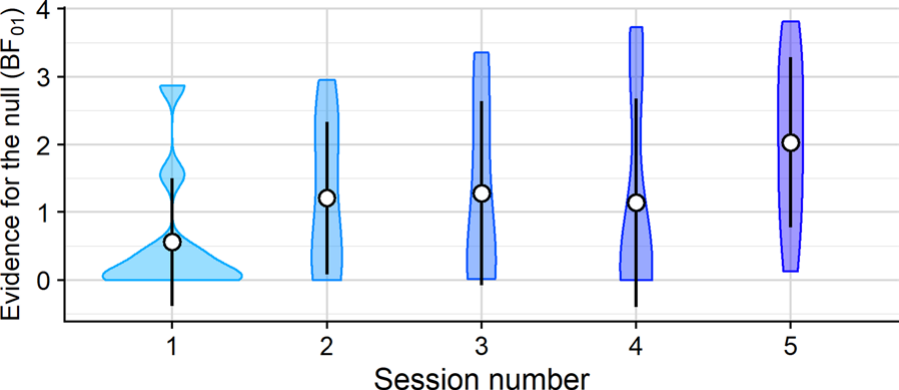
Violin plots showing Bayes factors for Bayesian *t*-tests comparing points earned by subjects in the incongruent search conditions over experimental sessions (*y*-axis) to points earned by ideal observers that used recent experience to search in the incongruent condition ($s =$ 1). White circles indicate the mean. Error bars represent $\pm 1$ standard deviation

Over sessions, evidence for the null was largely consistent for subjects in the congruent condition (Fig. 14). In sessions 1, 2, and 5, Bayes factors for 4 subjects (40%) supported the null hypothesis when compared to simulated search in the congruent condition ($M_{1} =$ 2.25, SD$_{1} =$ 0.51, $M_{2} =$ 2.99, SD$_{2} =$ 1.23, $M_{5} =$ 2.67, SD$_{5} =$ 1.04), whereas Bayes factors for 5 and 6 subjects (50% and 60%) supported the null in sessions 3 and 4, respectively ($M_{3} =$ 2.77, SD$_{3} =$ 1.06, $M_{4} =$ 2.83, SD$_{4} =$ 0.63). More subjects performed similarly to the ideal incongruent observer: in sessions 1 and 5, there were 6 subjects (60% and 67%^3^) whose Bayes factors supported the null ($M_{1} =$ 3.00, SD$_{1} =$ 1.05, $M_{5} =$ 2.74, SD$_{5} =$ 0.72), 7 subjects (70%) in sessions 3 and 4 ($M_{3} =$ 3.05, SD$_{3} =$ 0.54, $M_{4} =$ 3.07, SD$_{4} =$ 0.75), and 8 subjects (80%) in session 2 ($M_{2} =$ 2.22, SD$_{2} =$ 0.92).

*Incongruent human performance.*Overall, Bayes factors exceeded 1 for the ideal incongruent observer ($s =$ 1) in a total of 22 blocks (45%), and the average Bayes factor was 2.50 (SD = 0.84), suggesting searchers performed similarly to the ideal incongruent observer in the incongruent condition half of the time. Only 5 (10%) Bayes factors exceeded 1 ($M =$ 2.05, SD = 0.54) when human performance was compared to the ideal congruent observer, and 4 (8%) supported the null when subject data was compared performance of the ideal observer with partial world knowledge ($s =$ 60) in the incongruent condition ($M =$ 2.76, SD = 1.27). Consistent with human performance in the congruent condition, 0 Bayes factors supported the null when subject data was compared to simulated performance that relied solely on world knowledge in the incongruent condition ($s =$ 300).

When human data was compared to performance of the ideal incongruent observer ($s =$ 1), evidence favoring the null increased over sessions: Bayes factors for 2 subjects supported the null in session 1 ($M_{1} =$ 2.21, SD$_{1} =$ 0.93), 5 subjects (50%) in sessions 2 and 3 ($M_{2} =$ 2.15, SD$_{2} =$ 0.68, $M_{3} =$ 2.43, SD$_{3} =$ 0.92), 3 subjects (30%) in session 4 ($M_{4} =$ 3.35, SD$_{4} =$ 0.39), and 7 subjects (78%) in session 5 ($M_{5} =$ 2.51, SD$_{5} =$ 0.95). Bayes factors only supported the null when human data was compared to the ideal congruent observer twice (20%) in sessions 3 and 4 ($M_{3} =$ 2.20, SD$_{3} =$ 0.23, $M_{4} =$ 2.11, SD$_{4} =$ 0.94), and only one occurred in session 5 ($BF_{01} =$ 1.64). Two subjects performed similarly to the ideal incongruent observer that relied partially on world knowledge ($s =$ 60) in sessions 1 and 2 only ($M_{1} =$ 2.42, SD$_{1} =$ 1.93, $M_{2} =$ 3.10, SD$_{2} =$ 0.82), suggesting some subjects had learned some of the incongruent target locations in part during the first two sessions.

Overall, performance for most human searchers was comparable to ideal observers that learned target locations optimally, which suggests our subjects integrated world knowledge and recent experience in a near-optimal fashion. Human searchers became more similar to the ideal incongruent observer over experimental sessions, suggesting subjects learned to search in the semantically incongruent search environment over time. Human searchers did not perform as well as the ideal congruent observer in the semantically congruent experimental condition, despite the fact that the simulated searcher incurred point penalties for searching a location or switching rooms that were twice as high as those incurred by human searchers. These results suggest that human searchers learned from recent experience in ways that were near-optimal. That is, on the whole, both human and simulated searchers were able to learn to search effectively in the semantically incongruent search environment.

## Experiment 2: Discussion

Experiment 2 examined trade-offs between prior knowledge about scenes and recent experience during active visual search. We used the prior knowledge derived empirically in Experiment 1 to place targets in scenes and tested active search performance under conditions in which the locations of targets were either congruent with scene semantics, incongruent with respect to scene semantics, or random. A Bayesian ideal observer model was used to predict ideal search performance. Human search performance was compared to ideal observer simulations to evaluate how well searchers could learn the probabilistic structure of the task.

We found that, although search decisions were initially dominated by prior knowledge about scenes, approximately half of the searchers learned to prioritize information gained from recent experience in order to optimize search performance when prior knowledge was suboptimal, as evidenced performance for half of the subjects that was comparable to that of the Bayesian ideal observer according to Bayes factors for the comparison (44% on average, 78% by the last session).^4^ For those searchers who were able to prioritize recent experience over prior knowledge for scenes to guide search decisions, performance in the incongruent condition was never as good as in the congruent condition, indicating that it may be difficult to learn where to search for the target in a semantically incongruent environment. These findings show that there is some ability to learn to search incongruent locations in a near-optimal fashion using recent experience, and that the incongruent locations can be difficult to learn for certain targets.

Furthermore, our results demonstrate that human observers searched less effectively than simulated observers. Human observers earned fewer points on average in the congruent condition than simulated observers in the same condition, even though simulated observers paid higher penalties for searching a location and switching rooms. When human search performance was compared to the simulations, Bayes factors indicated that human observers in the congruent condition—when prior knowledge should have guided search—were more similar to the simulated observer in the *incongruent* condition, which should rely exclusively on recent experience. In other words, our results suggest that while human observers used prior knowledge to guide search decisions in our task, they did not do so perfectly—however, this is not surprising as human performance is generally not expected to be as good as that of ideal observers (Geisler 2011).

## General discussion

In the present work, we developed a novel search paradigm to investigate search decisions under the guidance of scene semantics and recent experience. We used a rating task (Experiment 1) to quantify prior semantic knowledge for the locations of common household objects in the specific scenes used in a search task (Experiment 2). The ratings were used to construct sampling distributions for target locations that were either congruent or incongruent with scene semantics, to select a subset of the objects to serve as targets in the search task, and to quantify scene semantics for the stimuli in a Bayesian ideal observer model. In Experiment 2, we used an active search task to investigate whether search decisions were guided by prior knowledge for scenes, recent experience, or a combination of both. Scenes were held constant and only the probability of target locations were manipulated. Our search task expanded on the search literature in three ways: (1) we determined semantically congruent and incongruent target locations empirically, (2) we investigated search in scenes where scene grammar was preserved, and (3) to determine whether scene semantics and recent experience are used optimally in visual search, we developed an ideal observer model that predicted ideal search performance when both information sources were used optimally. We compared subjects’ search performance against a Bayesian ideal observer model, which predicted optimal search behavior. While human search performance did improve over repeated experimental sessions, comparisons between the performance of human subjects and simulated observers revealed that subjects searched near-optimally on the whole, and revealed that about half of the subjects learned to prioritize recent experience to search effectively when target locations were incongruent with scene semantics. Overall, the results indicate that it is possible to negotiate between recent experience and prior knowledge for scenes to guide search in a near-optimal fashion when evidence that recent experience will facilitate search is strong.

Search for targets was not perfectly optimal for human subjects, as evidenced by fewer points earned in all conditions for human subjects than simulated searchers, despite the higher point penalties that were applied in the simulations. While human performance in general should not be equivalent to that of ideal observer models (Geisler 2011), there may be additional factors that explain the reported difference in performance. One possible explanation for these findings is that human observers may have required more observations than they were given ($N = 30$ trials/block) to infer which search environment they were in (congruent, incongruent, or random) and then employ the appropriate search strategy, whereas ideal observers were given hundreds of observations. It is possible that telling observers which search environment they are in (e.g., “Someone has hid objects from you in the scene.” for the incongruent condition) would result in more efficient search performance; however, given that explicit feedback about the target’s location after each trial did not affect search performance, explicit instruction may not have benefited the subjects. We leave further investigation of this possibility to future work.

It might seem surprising that searchers did not learn from recent experience more successfully, especially given that the search task was artificial. Rather than searching real-world scenes, which are processed more efficiently than drawings (Henderson and Ferreira 2004), subjects in the present study searched through computer-illustrated depictions of a room scene using sequences of mouse clicks to reveal the hidden contents of locations. Given the artificial nature of this task, one might expect subjects to quickly abandon their real-world prior knowledge in favor of recent experience, since target locations in both the congruent and incongruent conditions could be learned this way (as evidenced by the simulations), yet none of the subjects used such a strategy. While human searchers were able to learn to search in incongruent locations for the target to some extent, some searchers never showed that they could prioritize information gained from recent experience to guide search, even though most subjects were able to search effectively in the congruent condition. This suggests that semantic guidance was difficult to dismiss for those subjects, and that lower performance is best explained by the difficulty of learning to search in locations that conflict with scene semantics, and not by any lack of understanding of the task.

The difficulty of searching for the mug in the incongruent condition likely emerged from the difference in the number of locations that should be searched in the incongruent search environment as opposed to the corresponding congruent environment. That is, in the congruent environment, there was only one highly probable location (the cabinets) for the mug to be in, but in the incongruent environment, there were several highly probable locations (e.g., trash can, coat, backpack, couch). The fact that even the ideal observer was unable to learn to search for the mug in the incongruent condition suggests the incongruent target locations were not learnable. It is thus possible that both subjects and ideal observers would have learned more readily if the number of likely locations in the semantically congruent and incongruent sampling distributions were equal. The discrepancy between the number of probable locations under the semantically congruent and incongruent sampling distributions was a limitation of the current study. We intentionally selected targets for the active search task that were not strongly believed to be found in any one location (the keys and the batteries), and for a target that was strongly expected to be found in one location (the mug), to determine whether strong beliefs about where a target should be found would affect search decisions. Future research using a similar active search paradigm could address the aforementioned confound by testing only targets that are not strongly expected to occur in only one or two locations, and for which there are an equal number of probable sampling locations in the congruent and incongruent environments.

### Comparison to prior work

Our results provide additional evidence that incidental learning can occur in real-world scenes with intact scene semantics and grammar (Brockmole et al. 2006; Brockmole and Vo 2010; Brockmole and Henderson 2006a, b), even for meaningful targets (objects instead of letters), and expands on prior work by demonstrating that observers can use information learned from recent experience in a near-optimal fashion. In other respects, the findings of our active search task, in which search was carried out via sequences of mouse-clicks, differed from some of the results reported for gaze-directed search in which strong reliance on semantic knowledge was reported (Biederman et al. 1973; Castelhano and Heaven 2011; Vo and Wolfe 2013a, b). Differences between gaze-directed visual search and the present results could reflect the search modality (mouse click vs. gaze shift), the preservation of scene grammar in our display, or other task factors, such as the large number of observations (e.g., at least 60) that were required to learn.

The strategy of using recent experience rather than world knowledge to inform search decisions might be influenced by a desire to minimize search costs, where costs include the time or effort needed to search locations (Oliva et al. 2004; Kibbe and Kowler 2011; Rubenstein and Kowler 2018; Shenhav et al. 2017). In the search task, costs associated with search included the effort to operate the mouse, and the time delay between clicking a location and seeing its contents. Both of these costs provide incentives to improve search performance. For those subjects who did not learn in our search task, it is possible that the benefit of learning to search in incongruent target locations to improve performance was not worth the mental effort required to maintain information from recent experience in memory (Shenhav et al. 2017). Another possibility is that, for these searchers, search costs prompted the use of an encoding strategy that reduced processing demands during learning (e.g., by encoding summary statistics, not full distributions), but also limited accuracy (Dasgupta et al. 2018).

### Implications

Our results are consistent with two overarching ideas. First, search strategies, like other types of decision-making, involve the use of prior knowledge, and the strategy to rely on prior knowledge is, in most cases, an optimal one. Use of prior knowledge compensates for noisy input, and avoids taxing episodic memory (Steyvers et al. 2006; Hemmer and Steyvers 2009; Hemmer and Persaud 2014; Conci and Müller 2012) or motor resources (Santos and Kowler 2017). Second, search is Bayesian in that it was predicted by a Bayesian ideal observer model that included both prior knowledge and recent experience, and that recent experience informed search decisions (for all targets but the mug). Learning required multiple observations that contradicted prior knowledge, as evidenced by only two subjects learning to search in the incongruent condition effectively within the first session. One or a few occurrences of a target in an unusual place might not be sufficient to rely less on prior knowledge about scenes. For example, it may not be realistic for a subject to believe that mugs ought to be located in trash cans after only one observation. After accumulating sufficient evidence, however, we found that observers could use recent experience to guide search in a near-optimal fashion. It is unclear whether the individuals who did not learn from recent experience were unable to do so, if their evidence thresholds differed from those who did, or if the reward for doing so in the search task (points) was not sufficient to motivate learning. The latter, however, seems unlikely given that adaptive learning can take place with no reinforcement whatsoever (Kheifets and Gallistel 2012).

The formation of prior knowledge about scenes must involve, to some degree, the integration of episodic information. It is unclear when prior knowledge about scenes forms and solidifies, but there is some developmental evidence to suggest that they are early-emerging. At 24 months of age, children only notice semantic inconsistencies when inconsistent objects are visually salient, which suggests that scene semantics do not inform attention in scenes until later in development (Helo et al. 2017). By three years of age, children demonstrate sensitivity to the semantic context in a visual search paradigm (Öhlschläger and Vo 2016), suggesting that prior knowledge about scenes forms in the first four years of life. Semantic guidance seems to only be revealed in tasks where children’s eye-gaze is measured. Specifically, Öhlschläger and Vo (2016) found that although three-year-olds’ eye gaze is affected by semantic congruency, the same age group’s explicit response in a manual placing task was not. The performance contrast between tasks appears in other domains of child development research (e.g., physical reasoning, see Hood et al. 2000; theory of mind, see Baillargeon et al. 2010) and may result from task demands where an implicit response (i.e., eye-gaze) requires fewer cognitive resources than an explicit response (i.e., verbal response, manual response). It is possible that, for children, semantic guidance is influenced by the task costs.

The current findings also have implications for an atypical population where memory loss is a primary diagnostic characteristic (e.g., dementia). Healthy older adults rely on long-term semantic memory more heavily in visual search than younger adults do when new episodic information contradicts their world knowledge (Wynn et al. 2019). Individuals with dementia, such as patients with Alzheimer’s Disease, show deficits in both forms of memory (Hodges and Patterson 1995; see Nebes 1989 for review), which are often challenging to tease apart. The active search task may be able to discriminate between the two types of deficits by examining how patients with Alzheimer’s Disease perform in the semantically congruent condition (semantic recall) and the semantically incongruent condition (episodic recall) as compared to an ideal observer with varying dependence on the two. By distinguishing the type of memory deficit a patient has, clinicians would be better informed, and thereby, could implement a more targeted intervention to improve the patient’s cognitive processing. In addition, because our current findings suggest that neurotypical searchers learned over sessions, and previous research has shown that patients with Alzheimer’s Disease and amnesic patients are capable of implicitly learning new information across various domains: arbitrary letter configurations (Chun 2000; Chun and Phelps 1999), color-word associations (Musen and Squire 1993a), and word-pairs (Gabrieli et al. 1997; Moscovitch et al. 1986; Musen and Squire 1993b), individuals with memory deficits may do the same in both the semantically incongruent condition and the semantically congruent condition. Furthermore, research has found that the semantic memory impairment of patients with Alzheimer’s Disease appears to be caused by deficits in storage, and not a complete impairment in semantic memory itself (Hodges et al. 1992). This implication is especially relevant because implicit learning of semantic representations in this population may reduce the patients’ dependency on others to help them navigate the world.

### Conclusion

As in previous studies, searchers in the current experiment used prior semantic knowledge and recent experience to guide their search. After observing information that contradicted their prior semantic knowledge, searchers varied in their ability to integrate new episodic information into a probability distribution of target locations. Namely, some searchers learned quickly to prioritize recent experience (by the second session) while others learned at a slower rate (by the last session), or not at all. Even the observers who learned best did not achieve performance in the semantically incongruent condition that rivaled their use of prior knowledge in the semantically congruent condition. When target locations were incongruent with the semantics of the scene, scene semantics were not simply unhelpful (as was the case in Biederman et al. 1973; Neider and Zelinsky 2006; Henderson et al. 1999; Malcolm and Henderson 2009; Vo and Wolfe 2012, 2013b; Wu et al. 2014), they were actively detrimental to search performance because targets occurred in the *least* likely locations under guidance from their prior knowledge about scenes alone. As a result, observers who used scene semantics to inform their search decisions were set up to fail. Those who did not learn successfully may have relied more strongly on long-standing semantic beliefs, and therefore, recent experience that contradicted their beliefs may have been dismissed as events that were too unlikely to be repeated. The current findings are unable to differentiate between whether subjects constructed a new prior through learning from recent experience that targets occur in incongruent locations, or used scene semantics in an unconventional way (i.e., predicting incongruent target locations from locations that would be improbable based on scene semantics). Future work could compare search behavior for a target object with no previous semantic associations with the scene (e.g., the blicket) as a comparison: if observers are able to search equally well for a known object and a novel object in the incongruent condition, that would suggest observers constructed a new distribution of target location probabilities from scratch rather than using existing knowledge representations in an unconventional manner.

Our findings support the utility of Bayesian inference in visual search. The strategy to rely on prior knowledge for scene semantics to inform search in natural scenes is a rational one, which can be used efficiently and reliably in the majority of cases. Indeed, this strategy would be beneficial at best and harmless at worst 66.67% of the time in the present study: when target locations were consistent with scene semantics (semantically congruent), because semantic guidance would yield optimal inferences in this case, and when target locations were random, because no available strategy was better than another. It is also important to note that the incongruent sampling distribution was noisier than the congruent distribution in that there were more high probability locations for targets under the incongruent distribution than under the congruent distribution. The preference to rely on world knowledge about scene semantics therefore may also reflect a perfectly rational strategy to rely primarily on the more informative prior, even when doing so results in less efficient search behavior.

The current study has demonstrated that visual search is Bayesian: both prior knowledge and recent experience are used guide search in a near-optimal fashion. The degree to which recent experience can inform search decisions in the face of compelling evidence is influenced by how difficult it is to learn a statistical distribution from the evidence presented. An active search task like the one used in the current study provides a platform for understanding the nature of these trade-offs.

## Data Availability

All data, a link to a demo of the search task, and supplemental materials are available on the OSF: https://osf.io/swpum/

## Appendix: Simulation methods

### Design

Performance of the ideal observers was simulated with 3 levels of semantic congruence: (1) *semantically congruent:* target probabilities were completely congruent with the semantics of the scene, selected using the congruent sampling distribution obtained in Experiment 1 (see Eq. 1), (2) *random:* target location probabilities were uniform across all locations; and (3) *semantically incongruent:* target location probabilities were selected from the incongruent sampling distribution computed in Experiment 1 (Eq. 2), and thus were incongruent with scene semantics. The three levels of semantic congruence, the sampling distributions for the three targets (as found in Experiment 1), and the target location sampling method were all identical to those to be used in the search task.

Six different ideal observers were tested, varying in dependence on prior knowledge. The dependence was quantified as a quantity we named prior strength and denoted $s^k$ for ideal observer *k*. Low values for prior strength represented a strong reliance on recent experience, while high values represented a strong reliance on prior beliefs. Prior strength values (1, 30, 60, 90, 150, 300) were selected to simulate the full range of ideal observers.

### Procedure

The initial belief state of the ideal observers, shown in Eq. A.1, quantifies the probability of targets appearing in locations before any observations. This initial belief state was partly derived from the data obtained in Experiment 1 using Eq. A.2 as the mean probability of the Beta distribution. Eqs. A.3 and A.4 explain how the the shape and scale parameters of the Beta distribution’s parameters were scaled using the prior strength parameter, $s^k$.

A.1 $$\begin{aligned} P(\theta _{t,l}|w)~&=~{\mathrm {Beta}} (\alpha ^0_{t,l}, \beta ^0_{t,l}) \end{aligned}$$

A.2 $$\begin{aligned} \hat{p}^0_{t,l}~&=~\frac{\bar{L}_{t,l}}{7} \end{aligned}$$

A.3 $$\begin{aligned} \alpha ^0_{t,l}~&=~\hat{p}^0_{t,l}*s^k \end{aligned}$$

A.4 $$\begin{aligned} \beta ^0_{t,l}~&=~(1-\hat{p}^0_{t,l})*s^k \end{aligned}$$

As in the search task, a simulated searcher earned points for finding the target early within a trial. Points were set to the maximum possible (30) at the beginning of a trial, and were reduced as the searcher acted: 4 points were deducted for searching a location, and 2 points were deducted for switching between rooms. At the beginning of a trial, all 12 locations were unexplored. Locations to search were then sampled from the multinomial distribution (multinomial sampling) over all locations using Eq. A.5. By conducting multinomial sampling, the simulated searcher looked for the target in locations proportional to their probability. If the target was not found, the searched location was removed from consideration and the multinomial distribution recomputed. Each location could only be searched once. A trial terminated when either the target was found or the number of possible points earned reached 0.

A.5 $$\begin{aligned} P({\mathrm {Searching}}~l'|t')~&=~\frac{P(\theta _{t',l'}|r,w)}{\sum _{l''}P(\theta _{t',l''}|r,w)} \\&=~\frac{\hat{p}^0_{t',l'}}{\sum _{l''}\hat{p}^0_{t',l''}} \end{aligned}$$

The belief state of the searcher was updated at the end of each trial using Eq. A.6.^5^ Each trial is mathematically represented as a Bernoulli trial and thus combines with the Beta distribution of the belief to result in a new belief state. In practice, this requires updating the shape ($\alpha$) and scale ($\beta$) parameters of the distribution using the observation of success or observation of failure. To be more explicit, let the variable for the $i\hbox {th}$ trial be $t^i_{f,l}$ and it has value 1 if location *l* is searched and the target is found, otherwise if location *l* is searched and the target is not found, it has value 0. Shown in Eq. A.7, $\alpha ^i_{t,l}$ and $\beta ^i_{t,l}$ are updated from $\alpha ^{i-1}_{t,l}$ and $\beta ^{i-1}_{t,l}$ using $t^i_{f,l}$.

A.6 $$\begin{aligned} P(\theta ^i_{t,l}|r,w)~&= ~P(t^i_f|\theta ^{i-1}_{t,l}) P(\theta ^{i-1}_{t,l}|\alpha ^{i-1}_{t,l}, \beta ^{i-1}_{t,l}) \\ ~&=~P(t^i_f|\theta ^{i-1}_{t,l}) P(\theta ^{i-1}_{t,l}|\hat{p}^{i-1}_{t,l}, s^k) \end{aligned}$$

A.7 $$\begin{aligned} \alpha ^i_{t,l}~&=~\alpha ^{i-1}_{t,l}+t^i_f \\ \beta ^i_{t,l}~&=~\beta ^{i-1}_{t,l}+(1-t^i_f) \end{aligned}$$

With a little algebra, we rearrange the equation to construct a new $\hat{p}^i_{t,l}$ that takes into consideration the observation of success or failure for trial *i*, location *l*, and target *t*.^6^ This is shown in Eq. A.8.

A.8 $$\begin{aligned} \hat{p}^i_{t,l}~=~\frac{\hat{p}^{i-1}_{t,l}*s^k+t_{f,l}^i}{s^k+1} \end{aligned}$$

## Footnotes

1 Data collection took place in 2015.

2 All comparisons are available on the OSF.

3 One subject did not complete session 5.

4 See the OSF project repository for supplemental visualizations that show individual subjects’ performance.
